# Nitrogen‐Vacancy‐Rich VN Clusters Embedded in Carbon Matrix for High‐Performance Zinc Ion Batteries

**DOI:** 10.1002/advs.202308668

**Published:** 2024-03-13

**Authors:** Youcun Bai, Liang Luo, Wenliang Song, Shuaishuai Man, Heng Zhang, Chang Ming Li

**Affiliations:** ^1^ Institute for Materials Science and Devices School of Materials Science & Engineering Suzhou University of Science & Technology Suzhou 215011 P. R. China; ^2^ School of Chemistry and Chemical Engineering Chongqing University Chongqing 401331 China; ^3^ School of Materials and Chemistry University of Shanghai for Science and Technology Shanghai 200093 P. R. China; ^4^ School of Environment and Ecology Jiangnan University Wuxi 214122 P. R. China

**Keywords:** aqueous zinc ion battery, kinetics, nitrogen vacancies, quasi‐solid‐state battery, VN clusters

## Abstract

Vanadium nitride (VN) is a potential cathode material with high capacity and high energy density for aqueous zinc batteries (AZIBs). However, the slow kinetics resulting from the strong electrostatic interaction of the electrode materials with zinc ions is a major challenge for fast storage. Here, VN clusters with nitrogen‐vacancy embedded in carbon (C) (N_v_‐VN/C‐SS‐2) are prepared for the first time to improve the slow reaction kinetics. The nitrogen vacancies can effectively accelerate the reaction kinetics, reduce the electrochemical polarization, and improve the performance. The density functional theory (DFT) calculations also prove that the rapid adsorption and desorption of zinc ions on N_v_‐VN/C‐SS‐2 can release more electrons to the delocalized electron cloud of the material, thus adding more active sites. The N_v_‐VN/C‐SS‐2 exhibits a specific capacity and outstanding cycle life. Meanwhile, the quasi‐solid‐state battery exhibits a high capacity of 186.5 mAh g^−1^, ultra‐high energy density of 278.9 Wh kg^−1^, and a high power density of 2375.1 W kg^−1^ at 2.5 A g^−1^, showing excellent electrochemical performance. This work provides a meaningful reference value for improving the comprehensive electrochemical performance of VN through interface engineering.

## Graphical abstract

Vanadium nitride clusters with nitrogen‐vacancy embedded in carbon (N_v_‐VN/C‐SS‐2) are prepared for the first time. The nitrogen vacancies promote faster reaction kinetics and provide more active sites, and the carbon matrix improves the conductivity and effectively ensures the activity of the VN cluster. So, N_v_‐VN/C‐SS‐2 exhibits high specific capacity, excellent rate capability, and satisfactory cycle stability.

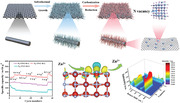

## 1 Introduction

In recent years, electronic products have affected people's daily lives, and batteries have naturally become common energy storage devices.^[^ ^1^, ^2^, ^3^ ^]^ As we all know, lithium‐ion batteries have been very common, but there are still many shortcomings, such as harsh production environment, high cost, unsafe organic electrolyte, and so on.^[^ ^4^, ^5^ ^]^ On the contrary, AZIBs have attracted much attention due to their low cost, high safety, and good electrochemical performance.^[^ ^6^, ^7^ ^]^ However, the properties of electrode materials affect its further development.^[^ ^8^ ^]^ Therefore, improving the performance of cathode materials is an important step in the development of AZIBs.^[^ ^9^ ^]^

Vanadium‐based materials show excellent electrochemical performance due to the multiple oxidation states of vanadium and open crystal structure and become an attractive cathode material for AZIBs.^[^ ^10^, ^11^ ^]^ For example, Liu et al. prepared PANI‐intercalated V_2_O_5_ electrode, this not only creates a large interlayer spacing (13.90 Å) between the V‐O layers, but also provides an accelerated channel for the diffusion of Zn^2+^, thus exhibiting excellent electrochemical properties.^[^ ^12^ ^]^ In addition, besides oxides, some vanadium compounds (Zn_0.25_V_2_O_5_·0.85H_2_O,^[^ ^13^ ^]^ Cu_3_V_2_O_7_(OH)_2_·2H_2_O,^[^ ^14^ ^]^ (NH_4_)_2_V_6_O_16_·1.5H_2_O,^[^ ^15^ ^]^ Mg_0.1_V_2_O_5_·H_2_O,^[^ ^16^ ^]^ and NaV_6_O_15_^[17]^) and vanadium chalcogenides (VS_2_^[18]^/VSe_2_^[19]^) have been reported to have good energy storage properties for Zn^2+^. However, these cathode materials are still facing some problems such as easy to fall off and dissolve, structural instability, and so on, which further limit their development.^[^ ^20^ ^]^ It is still a challenge to find a cathode material with excellent performance and suitable for Zn^2+^ ion intercalation and deintercalation.^[^ ^21^ ^]^

Compared with metal oxides, transition metal nitrides (TMNs) have attracted more attention because of their good conductivity, structural/chemical stability, and strong mechanical strength,^[^ ^22^ ^]^ among which VN is a potential electrode material with metalloid high conductivity, pseudocapacitance, high density, and platinum‐like catalytic properties.^[^ ^23^ ^]^ Zhao et al reported a VN hollow sphere material prepared by template‐assisted strategy, which showed enhanced performances in lithium‐ion batteries.^[^ ^24^ ^]^ Wei et al prepared VN by one‐step ammoniation of V_2_C MXene, which showed good electrochemical performance in the process of sodium storage.^[^ ^25^ ^]^ Although VN has made some progress in the field of energy storage, the problems of slow reaction kinetics and unstable cycle performance in the electrochemical test process of VN materials have not been improved. It is proved that compounding with carbon materials is an effective method to improve cycle stability.^[^ ^26^ ^]^ In addition, it is exciting that the construction of vacancy structure has been proven to be an effective method to improve the electrochemical performance due to its high conductivity, fast kinetics, and abundant active sites, which will be beneficial to improve the overall performance of the battery.^[^ ^27^, ^28^ ^]^ Liu et al introduced Cu ions and O vacancies into Co_3_O_4_ nanocrystals simultaneously and realized significant improvement in specific capacity and rate capability due to the enhanced conductivity.^[^ ^29^ ^]^ Although a large number of literatures have proved that the introduction of vacancies in electrode materials can effectively improve the electrochemical performance, the change of electrochemical reaction mechanism caused by vacancies is still ambiguous. Most importantly, these surface modification methods are not suitable for large‐scale production of high‐performance electrode materials because of their complex preparation and high cost.

Here, we construct a nitrogen vacancy‐rich self‐supporting N_v_‐VN/C‐SS‐2 nanorod cathode. The introduction of appropriate nitrogen vacancies promotes faster reaction kinetics and provides more active sites, and the carbon matrix improves the conductivity and effectively ensures the activity of the VN cluster. Importantly, through the study of the electrochemical reaction mechanism, it is found that the introduction of nitrogen vacancies can effectively improve the electrochemical polarization. As a result, N_v_‐VN/C‐SS‐2 exhibits high specific capacity (257 mAh g^−1^ at 0.2 A g^−1^), excellent rate capability (301 mAh g^−1^ at 5 A g^−1^), and satisfactory cycle stability (227 mAh g^−1^ after 1000 cycles at 4 A g^−1^). The quasi‐solid N_v_‐VN/C‐SS‐2//Zn battery also has high specific capacity, and high energy density, and the flexible quasi‐solid device can adapt to various extreme environments.

## 2 Results and Discussion

### 2.1 Material Characterization

**Figure 1:**
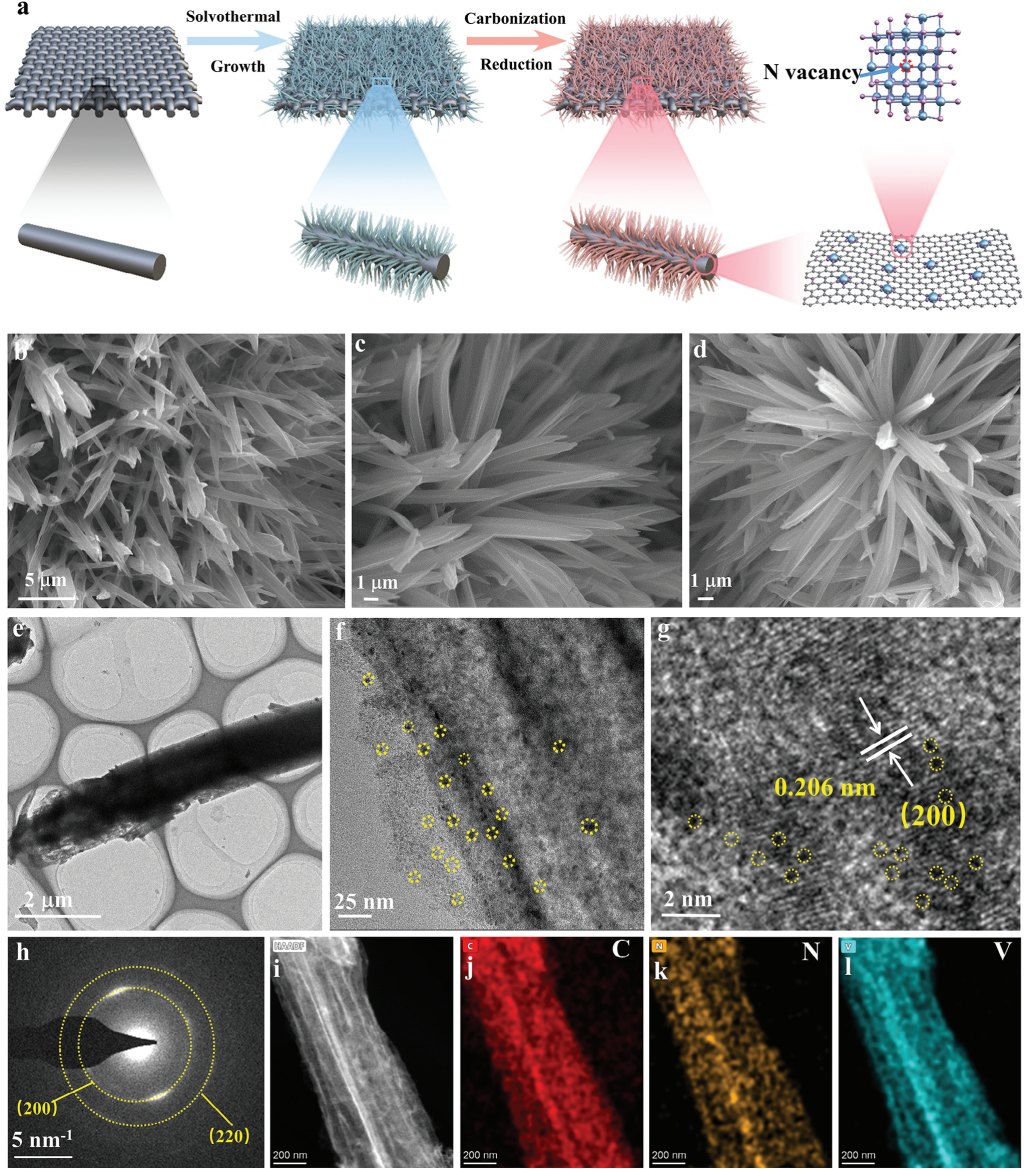
a) Schematic diagram of the preparation of N_v_‐VN/C‐SS‐2, b) SEM of precursor, c) N_v_‐VN/C‐SS‐1, and d) N_v_‐VN/C‐SS‐2, e) TEM, f, g) HRTEM, h) SAED images, i–l) HAADF‐STEM mapping of N_v_‐VN/C‐SS‐2.

An illustrative procedure for the synthesis of the nitrogen‐vacancy‐rich N_v_‐VN/C‐SS‐2 stainless steel (SS) support electrode material by the reduction strategy is shown in **Figure** **1**a. First, vanadium ions are coordinated with terephthalic acid in the solvothermal process. With the subsequent high‐temperature nitridation process in a reducing atmosphere, the organic matter is decomposed to finally generate the composite electrode material with vanadium nitride embedded in a carbon matrix. Figure S1 (Supporting Information) and Figure 1b show a scanning electron microscope (SEM) image of a precursor sample with a large number of uniform nanorods grown on the stainless steel (SS). The micromorphology of N_v_‐VN/C‐SS‐1 (Figure 1c; Figure S2, Supporting Information) and N_v_‐VN/C‐SS‐2 (Figure 1d; Figure S3, Supporting Information) did not change significantly after nitridation, while the morphology of N_v_‐VN/C‐SS‐3 (Figure S4, Supporting Information) showed some damage after nitridation for 3 h, the breakdown of the structure may lead to degradation of the electrochemical properties. The micromorphology of the nanorods of N_v_‐VN/C‐SS‐2 was further investigated by transmission electron microscopy (TEM) (Figure 1e). The nanorod micromorphology of N_v_‐VN/C‐SS‐2 can be clearly observed, which is consistent with the result of SEM. At the same time, As can be seen from the high‐resolution TEM image (HRTEM), the VN clusters (yellow circle) of ≈2 nm are uniformly dispersed on the carbon matrix (Figure 1f), and the highly uniform dispersion of VN with high theoretical capacity ensures high specific capacity.^[^ ^30^, ^31^, ^32^ ^]^ In addition, it can be seen from Figure 1g that the interplanar spacing of 0.206 nm corresponds to the (200) crystal plane of VN (JCPDS 73–2038).^[^ ^23^ ^]^ Due to the small size of VN and the fact that it is embedded in carbon, this results in blurred lattice striations as shown in the yellow box in the figure (Figure 1g). The corresponding selected area electron diffraction (SAED) pattern (Figure 1h) also confirmed the weak crystallinity of N_v_‐VN/C‐SS‐2, this may be due to the small size of the VN embedded in the carbon matrix and the presence of vacancies that affect its crystallinity. Furthermore, TEM mapping shows the uniform distribution of V, C, and N elements in the N_v_‐VN/C‐SS‐2 electrode material (Figure 1i–l).

**Figure 2:**
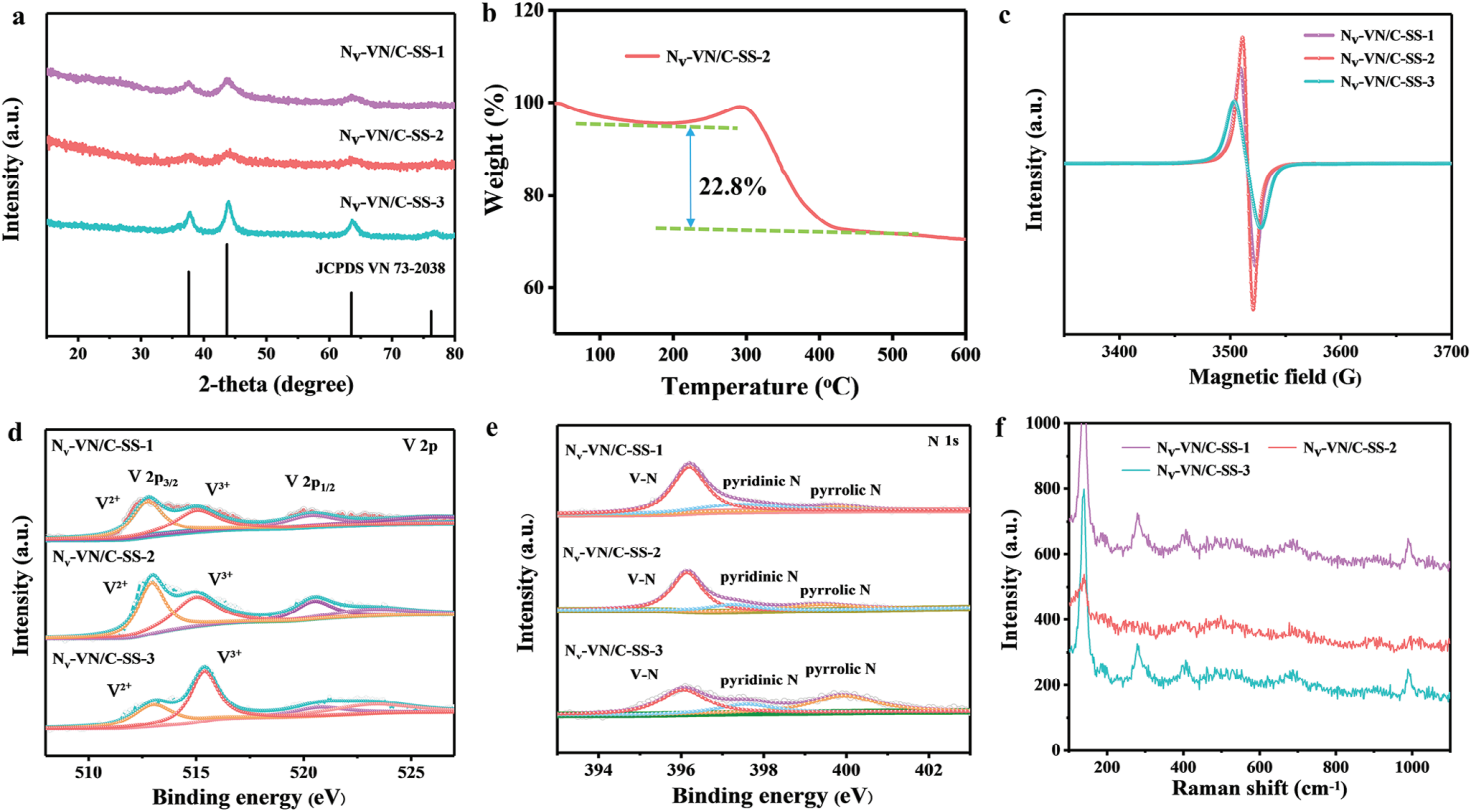
a) XRD patterns of N_v_‐VN/C‐SS‐1, N_v_‐VN/C‐SS‐2, and N_v_‐VN/C‐SS‐3, b) TGA of N_v_‐VN/C‐SS‐2, c) EPR spectra, and d) XPS spectra of N_v_‐VN/C‐SS‐1, N_v_‐VN/C‐SS‐2, and N_v_‐VN/C‐SS‐3: V 2p, e) N 1s, f) Raman spectrum of N_v_‐VN/C‐SS‐1, N_v_‐VN/C‐SS‐2, and N_v_‐VN/C‐SS‐3.

The X‐ray powder diffraction (XRD) patterns of N_v_‐VN/C‐SS‐1, N_v_‐VN/C‐SS‐2, and N_v_‐VN/C‐SS‐3 are shown in **Figure** **2**a, and the XRD diffraction peaks of all samples can match the standard card of VN (JCPDS 73–2038) and have no impurity peaks, which proves that the VN is successfully synthesized.^[^ ^23^ ^]^ In addition, no diffraction peak of carbon was observed, which may be caused by low carbon content. Based on the results of thermogravimetric analysis (TGA), the carbon content in Nv‐VN/C‐SS‐2 was calculated to be 22.8% (Figure 2b). The successful introduction of N vacancies was further demonstrated by electron paramagnetic resonance (EPR) testing, all samples showed symmetric signals at g≈2.008 (Figure 2c), indicating the presence of nitrogen vacancies in the crystal lattice.^[^ ^33^ ^]^ Meanwhile, it can be observed that the EPR intensity of N_v_‐VN/C‐SS‐2 is stronger than that of N_v_‐VN/C‐SS‐1 and N_v_‐VN/C‐SS‐3, which means that more vacancies are created in the crystal of N_v_‐VN/C‐SS‐2. This is because the precursor will be gradually converted into VN under the action of C_3_H_6_N_6_, and some oxygen atoms may occupy the position of N in VN after 1 h of reaction, and with the prolongation of time, the oxygen atoms will be gradually replaced by N so that N content is increase and the vacancy is reduced. The surface elemental composition of the three samples was further studied by X‐ray photoelectron spectroscopy (XPS). The high‐resolution V 2p XPS spectrum is shown in Figure 2d. Wherein peaks at 513.5 and 515.4 eV are assigned to V^2+^ and V^3+^, respectively.^[^ ^34^ ^]^ Interestingly, with the increase of N vacancy content, the content of the V^2+^ state increases from 57.6% (N_v_‐VN/C‐SS‐3) and 65.4% (N_v_‐VN/C‐SS‐1) to 72.5% (N_v_‐VN/C‐SS‐2). The nitrogen vacancies effectively activate V^2+^, and the low valence ions are beneficial to the improvement of conductivity.^[^ ^29^ ^]^ The high‐resolution XPS spectrum of N 1s is shown in Figure 2e, where a peak at 396.2 eV is V─N bond, and the two peaks near 397.8 and 399.9 eV are pyridinic N and pyrrolic N.^[^ ^35^ ^]^ Figure 2f is the Raman spectrum of the three samples, and the main characteristic peaks of VN appear at 139.4, 280.7, and 406.6 cm^−1^ for all the samples.^[^ ^23^ ^]^ Importantly, the N_v_‐VN/C‐SS‐2 shows the weakest peak intensity, this proves that the abundance of nitrogen vacancies weakens the vibrational strength.^[^ ^36^ ^]^

### 2.2 Electrochemical Performance

**Figure 3:**
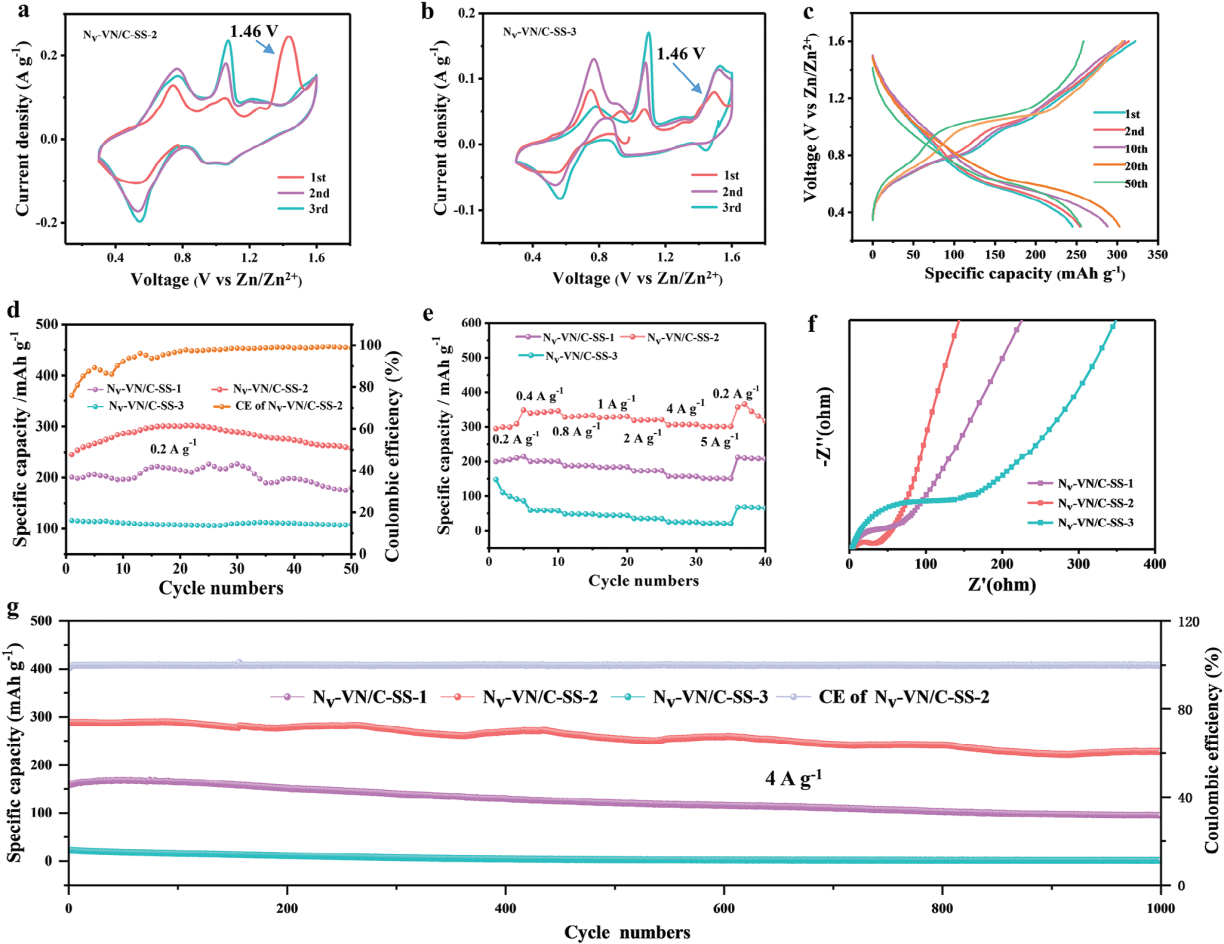
a) CVs curves of N_v_‐VN/C‐SS‐2 and b) N_v_‐VN/C‐SS‐3, c) charge‐discharge profiles of N_v_‐VN/C‐SS‐2, d) cycle performances, e) rate performances, f) impedance plots, and g) long cycle life performances of N_v_‐VN/C‐SS‐1, N_v_‐VN/C‐SS‐2, and N_v_‐VN/C‐SS‐3.

The electrochemical performance of N_v_‐VN/C‐SS‐1, N_v_‐VN/C‐SS‐2, and N_v_‐VN/C‐SS‐3 electrodes was compared by using a typical button cell. **Figure** **3**a,b shows a cyclic voltammetry (CV) curve of the first three cyclic of N_v_‐VN/C‐SS‐2 and N_v_‐VN/C‐SS‐3 at 0.1 mV s^−1^, respectively. And the CV curve for N_v_‐VN/C‐SS‐1 is shown in Figure S5 (Supporting Information). It can be seen that there is a strong oxidation peak near 1.46 V in the first turn of all samples, which may be caused by the phase transition, it is worth noting that the strong oxidation peak of N_v_‐V/C‐SS‐3 does not disappear after three cycles, which may be related to its poor reversibility and strong polarization.^[^ ^37^ ^]^ This may indicate that the formation of vacancies can significantly promote the reversible reaction because more low‐valent metal ions are produced, which is more conducive to the electrochemical reaction. In addition, the two pairs of redox peaks at ≈1.07/0.92 and 0.76/0.53 V in the last two cycles may be related to the stepwise intercalation and deintercalation of Zn^2+^.^[^ ^6^ ^]^ The charge/discharge curve of N_v_‐VN/C‐SS‐2 is shown in Figure 3c, the significant charging and discharging platforms are positioned at ≈1.12 and 0.65 V, respectively, which is consistent with the CV test result. As a result, a high discharge specific capacity of 257 mAh g^−1^ can be achieved (50 cycles, 0.2 A g^−1^) (Figure 3d), which is higher than most of the currently reported AZIBs^[^ ^10^, ^14^, ^17^, ^18^, ^19^, ^38^, ^39^, ^40^, ^41^, ^42^ ^]^ (Table S1, Supporting Information). In contrast, N_v_‐VN/C‐SS‐1, and N_v_‐VN/C‐SS‐3 exhibit poor specific capacity, the specific capacities are 180 and 108 mAh g^−1^, respectively. In addition, as shown in (Figure 3e), the specific capacities at 0.2, 0.4, 0.8, 1, 2, 4, and 5 A g^−1^ were 299, 341, 330, 328, 319, 306, and 301 mAh g^−1^, showing excellent rate performance. N_v_‐VN/C‐SS‐1 and N_v_‐VN/C‐SS‐3 also exhibit relatively poor rate performance, which can be attributed to the fact that the abundance of vacancies in N_v_‐VN/C‐SS‐2 promotes the electrochemical reaction kinetics. This conclusion is further confirmed by electrochemical impedance spectroscopy (Figure 3f; Figure S3 and Table S2, Supporting Information). As can be seen from the embedded equivalent circuit diagram, N_v_‐VN/C‐SS‐2 (33.82 Ω, 0.3026 Ω) has a lower *R*_ct_ value and *Z*_w_ than N_v_‐VN/C‐SS‐1 (60.52 Ω, 0. 0.3106 Ω) and N_v_‐VN/C‐SS‐3 (132.51 Ω, 0.3502 Ω). Distinctly, the N_v_‐VN/C‐SS‐2 has the fastest ion transfer rate and shows excellent electrochemical performance. Figure 3g shows the long cycle performance of N_v_‐VN/C‐SS‐1, N_v_‐VN/C‐SS‐2, and N_v_‐VN/C‐SS‐3 at high current density 4 A g^−1^. The collapse of electrode material structure and the decrease of active sites lead to the gradual attenuation of specific capacity during the process of electrochemical reaction. N_v_‐VN/C‐SS‐2 still keeps the specific capacity of 227 mAh g^−1^ after 1000 cycles; however, the specific capacity of N_v_‐VN/C‐SS‐1 and N_v_‐VN/C‐SS‐3 were only 95 and 1.5 mAh g^−1^ after 1000 cycles under the same test condition, respectively. The excellent cycling stability also indicates that the vacancy structure of N_v_‐VN/C‐SS‐2 can withstand long periods of rapid current surges. Moreover, this excellent electrochemical performance is due to the stable embedding and high dispersion of VN clusters in C, which effectively avoids the problem of agglomeration and shedding of nanomaterials, and ensures the effective utilization of active substances. In addition, the C Matrix ensures high conductivity and structural stability. Importantly, the abundant nitrogen vacancies effectively increase the active sites, increase the zinc ion diffusion rate, and improve the electrochemical performance. NV‐VN/C‐SS‐2 was in situ grown on stainless steel by the method of solution heat. Compared with powder samples, it can effectively improve the loading capacity in large‐scale applications, but it still has the problems of capacity fading and conductivity weakening, which still needs the efforts of many researchers in the future.

**Figure 4:**
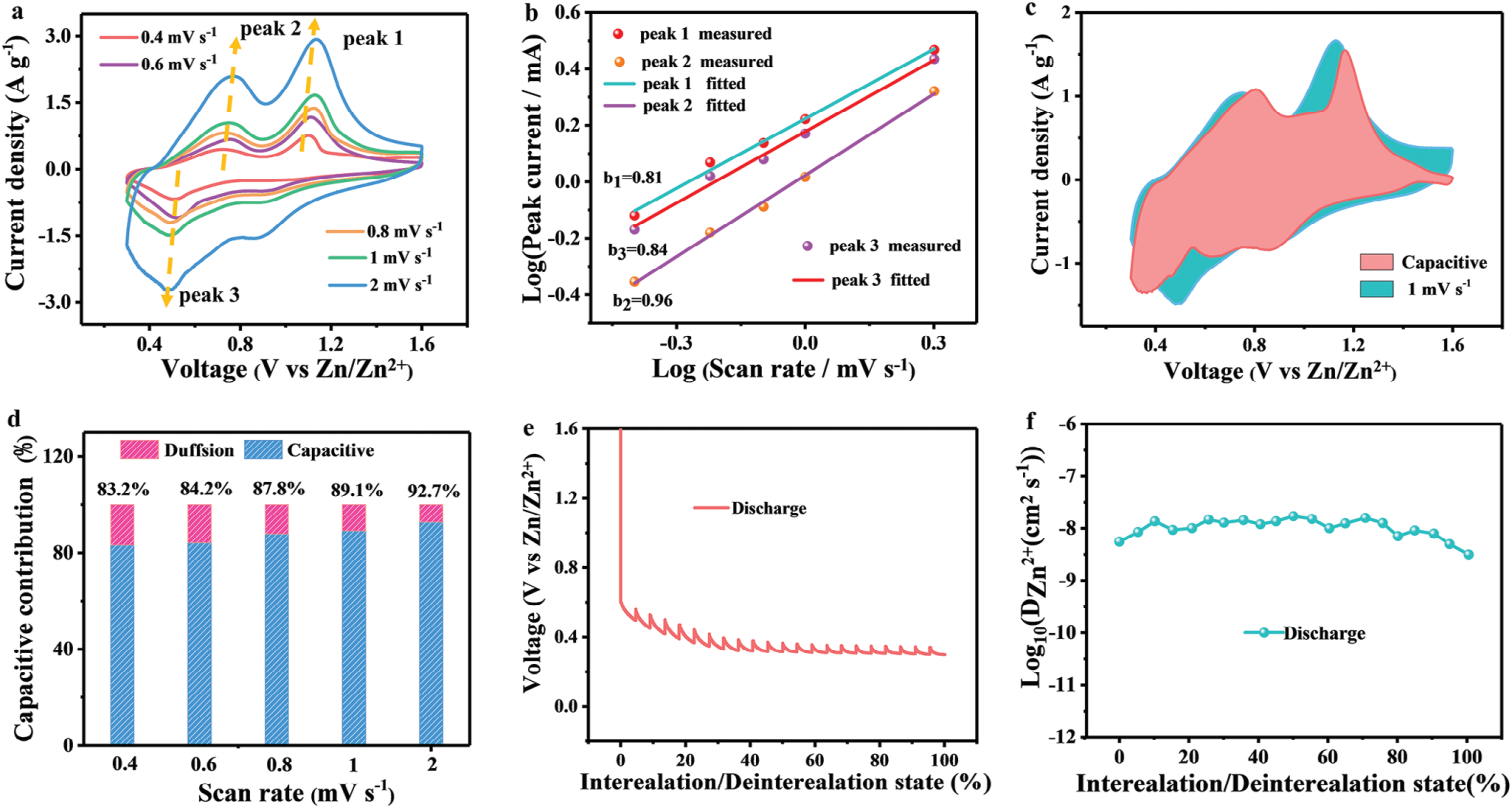
a) CV curves, b) Log (*i*) versus log (*v*) plots, c) capacitive contribution at 1 mV s^−1^, d) contribution ratio of capacitance and diffusion‐controlled, e) GITT curves, f) Zn^2+^ diffusion coefficient.

In order to verify the role of vacancy, the cyclic voltammetry tests were carried out at different scan rates. **Figure** **4**a shows the CV curves of N_v_‐VN/C‐SS‐2 at various scan rates. It can be seen that the peak area increases and the shape does not change with the increase of scan rate. In general, the scan rate (*v*) and the corresponding peak current (*i*) can be described by a power law *(i* = *av^b^)*.^[^ ^28^ ^]^ As can be seen from Figure 4b, the b values of peak 1 to peak 3 are 0.81, 0.96 and 0.84, respectively. The results show that for this electrode material, the b value is closer to 1.0, which is mainly controlled by the capacitance.^[^ ^30^ ^]^ Meanwhile, the CV curves of N_v_‐VN/C‐SS‐1 and N_v_‐VN/C‐SS‐3 are compared under the same test conditions, as shown in Figures S7 and S8 (Supporting Information), respectively. Obviously, both N_v_‐VN/C‐SS‐1 (Figure S7a, Supporting Information) and N_v_‐VN/C‐SS‐3 (Figure S8a, Supporting Information) show poor reversibility compared to N_v_‐VN/C‐SS‐2. This further proves that nitrogen vacancies play an important role in improving the reversibility of the reaction.^[^ ^32^ ^]^ The b values of N_v_‐VN/C‐SS‐1 and N_v_‐VN/C‐SS‐3 are also shown in Figures S7b and S8b (Supporting Information), respectively. For N_v_‐VN/C‐SS‐1, the b values of peak 1 to peak 3 are 0.61, 0.97, and 0.81, and the b values of N_v_‐VN/C‐SS‐3 are 0.68, 0.93, and 0.89, respectively. We also calculated the exact capacitance contributions for N_v_‐VN/C‐SS‐1, N_v_‐VN/C‐SS‐2, and N_v_‐VN/C‐SS‐3 using the following equation:

(1) $$i=k_{1}v+k_{2}v^{1/2}$$

As shown in Figure 4c, N_v_‐VN/C‐SS‐2 has a capacitance contribution of 89.1% at a scan rate of 1 mV s^−1^, which is higher than the contributions of N_v_‐VN/C‐SS‐1 (84.5%) (Figure S7c, Supporting Information) and N_v_‐VN/C‐SS‐3 (81.1%) (Figure S8c, Supporting Information). This means that the introduction of nitrogen vacancies effectively increases the reaction's active surface and obtains a more rapid surface capacitance reaction, which is conducive to improving the rate capability of N_v_‐VN/C‐SS‐2 electrode material.^[^ ^33^ ^]^ The capacitance contributions of N_v_‐VN/C‐SS‐1, N_v_‐VN/C‐SS‐2, and N_v_‐VN/C‐SS‐3 at different scan rates are shown in Figure 4d, Figures S7d and S8d (Supporting Information), respectively.

In order to further explore the role of nitrogen vacancies, the reaction kinetics of N_v_‐VN/C‐SS‐1, N_v_‐VN/C‐SS‐2, and N_v_‐VN/C‐SS‐3 were compared by galvanostatic intermittent titration technique (GITT). As shown in Figure 4e,f, N_v_‐VN/C‐SS‐2 obtained a larger Zn^2+^ diffusion coefficient (D _Zn_^2+^≈1.21×10^−8^) during discharge, indicating rapid reaction kinetics. On the contrary, N_v_‐VN/C‐SS‐1 (D_Zn_^2+^≈3.16×10^−9^) (Figure S7e,f, Supporting Information) and N_v_‐VN/C‐SS‐3 (D_Zn_^2+^≈5.42×10^−10^) (Figure S8e,f, Supporting Information) with lower content of vacancies show slower diffusion behavior of Zn^2+^. Based on the above studies, it is demonstrated that nitrogen vacancies can improve the reaction kinetics and correspondingly increase the capacitance contribution. Therefore, the introduction of nitrogen vacancies is of great significance to improve the electrochemical performance.

**Figure 5:**
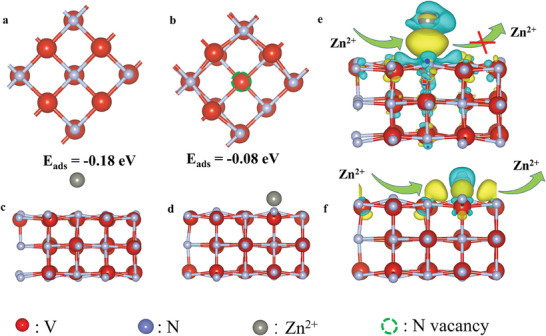
a,b) The DFT calculation. The model of VN without/with vacancies, c,d) the adsorption/desorption of Zn^2+^ on the N_v_‐VN/C‐SS‐3 and N_v_‐VN/C‐SS‐2, charge density for Zn^2+^ storage/release on N_v_‐VN/C‐SS‐3 e) and f) N_v_‐VN/C‐SS‐2.

To further understand the effect of nitrogen vacancies on the electronic properties of N_v_‐VN/C‐SS‐2 nanorods, we performed DFT calculations based on the model of VN without/with vacancies (**Figure** **5**a,b). Compared with the low adsorption energy of N_v_‐VN/C‐SS‐3 (−0.18 eV, Figure 5c), the vacancy‐rich N_v_‐VN/C‐SS‐2 has more suitable adsorption energy (−0.08 eV, Figure 5d), which makes it easier for Zn^2+^ to be reversibly adsorbed and desorbed on the N_v_‐VN‐C‐SS‐2 surface.^[^ ^43^ ^]^ This means that once occupied electrochemical active sites can be reused in a short time, which is further evidenced by differential charges. Compared with N_v_‐VN/C‐SS‐3 (Figure 5e), the rapid adsorption/desorption of Zn^2+^ on N_v_‐VN/C‐SS‐2 (Figure 5f) can release more electrons to the delocalized electron cloud of the material, thereby increasing the reversible capacity.^[^ ^44^ ^]^

**Figure 6:**
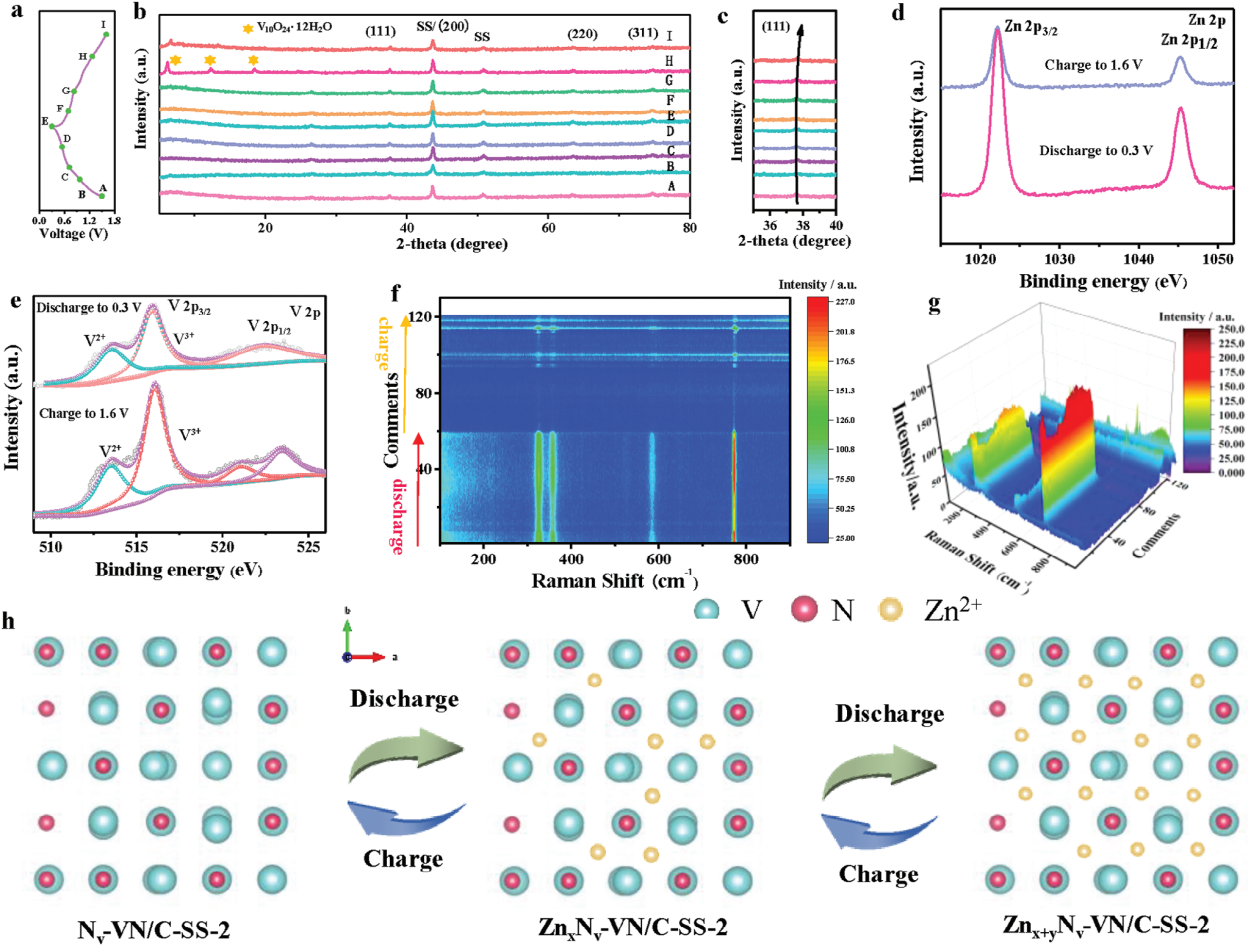
a) The charge–discharge curve, b) XRD patterns, c) magnified XRD patterns of b, d) Zn 2p spectra, e) V 2p spectra, f,g) the contour plot and the 3D mapping surface of the in situ Raman spectrum, h) mechanism illustration of zinc ion in the N_v_‐VN/C‐SS‐2.

The unique activation process and excellent electrochemical properties of the N_v_‐VN/C‐SS‐2 arouse our interest in further revealing the mechanism of Zn^2+^ storage. The phase and position changes during charging and discharging (**Figure** **6**a) were monitored by ex situ XRD measurements. As shown in Figure 6b,c, during the discharging process, no new diffraction peak was observed except that the diffraction peak of the (111) plane shifted to a lower angle due to the intercalation of Zn^2+^,^[^ ^20^ ^]^ while during the charging process, a diffraction peak of V_10_O_24_⋅12H_2_O (JCPDS 25–1006) appeared when the voltage was charged to 1.45 V, which corresponds to the CV curve. Importantly, the characteristic peak almost returns to the original position when charging to 1.6 V, which reveals a high degree of reversibility during charging/discharging.^[^ ^9^ ^]^ High‐resolution XPS analysis further revealed the change in the surface valence state of elements. The XPS spectra of Zn 2p at different potentials are shown in Figure 6d, and it can be seen that there is a stronger characteristic peak at 0.3 V due to the intercalation of Zn^2+^.^[^ ^44^ ^]^ The V 2p XPS spectrum is shown in Figure 6e. The peaks at ≈513.5 and 515.6 eV are characteristic peaks of V^2+^ and V^3+^, respectively.^[^ ^34^ ^]^ Due to the intercalation/deintercalation of Zn^2+^, the electrode has the strongest V^3+^ in the fully charged state and the strongest V^2+^ in the fully discharged state. In situ Raman spectroscopic analysis of the N_v_‐VN/C‐SS‐2 at various voltages during charge and discharge is shown in Figure 6f,g. It can be seen that from the contour plot (Figure 6f) and the 3D mapping surface (Figure 6g) of the in‐situ Raman spectrum, there are obvious vibrational characteristic peaks of VN at 325, 359, 586, and 772 cm^−1^, respectively. In the whole charge‐discharge process, all the peaks showed similar trends, and the peak intensity did not change significantly in the discharge process, indicating that the structure is stable, while in the process of charging, the intensity of the characteristic peak weakened significantly, until close to the full charge state (close to 1.6 V), the obvious characteristic peak was observed, which may be affected by by‐products. The peak of N_v_‐VN/C‐SS‐2 was observed again with the disappearance of the by‐products. The results of Raman study were consistent with those of ex‐situ XRD. Figure S9 (Supporting Information) also shows the Raman spectra of N_v_‐VN/C‐SS‐2 under different voltages, and the change in peak intensity can be seen more intuitively. The above research results combined with the reaction kinetics in Figure 4 can confirm that the energy storage mechanism of Zn^2+^ in N_v_‐VN/C‐SS‐2 is the adsorption and desorption of Zn^2+^ on N_v_‐VN/C‐SS‐2 surfaces and the intercalation and deintercalation of Zn^2+^, in which the deintercalation mechanism of Zn^2+^ is shown in Figure 6h. Besides, TEM mapping (Figure S10, Supporting Information) indicates that N_v_‐VN/C‐SS‐2 can still ensure the uniform distribution of N, V, C, and Zn elements after 1000 cycles at 4 A g^−1^, which proves the stability of the structures.

**Figure 7:**
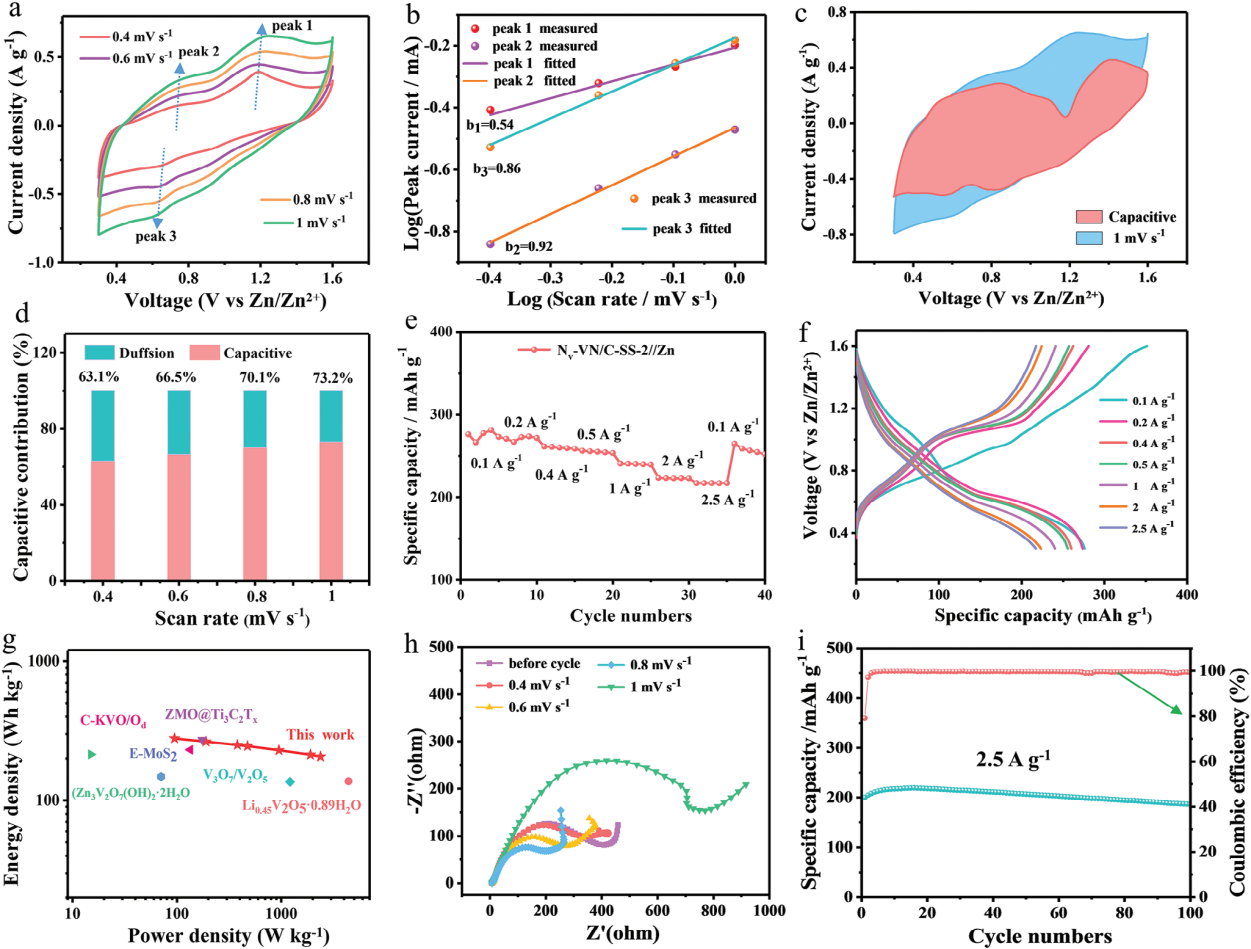
a) CVs curves at different scan rates, b) log(*i*) versus log(*v*) plots, c) CV area contributed by capacitance at 1 mV s^−1^, d) contribution ratio of capacitance at different scan rates, e) rate performance, f) charge–discharge curves at various current densities, g) comparison of energy density and power density with those reported in literature, h) EIS spectra, and i) cycle performance.

In order to evaluate the storage capacity of Zn^2+^ in a quasi‐solid‐state (QSS) N_v_‐VN/C‐SS‐2//Zn battery, the coin cell was assembled by using N_v_‐VN/C‐SS‐2 as cathode, the prepared gel electrolyte as separator and electrolyte, and zinc foil as anode. Firstly, the cyclic voltammetry (CV) curves of QSS N_v_‐VN/C‐SS‐2//Zn battery at different scan rates were tested (**Figure** **7**a), and two pairs of redox peaks were observed, indicating that the process of Zn^2+^ intercalation and deintercalation is divided into two steps,^[^ ^13^ ^]^ this is consistent with previous studies of aqueous systems. The shape of the CV curve remained well with an increasing scan rate, indicating the structural stability of the N_v_‐VN/C‐SS‐2 cathode.^[^ ^40^ ^]^ Besides, to reveal the energy storage mechanism of Zn^2+^ in the QSS N_v_‐VN/C‐SS‐2//Zn battery, we calculated the b value. As shown in Figure 7b, the values of b2 and b3 are both close to 1, indicating that there is mainly capacitance control.^[^ ^6^ ^]^ The contribution of capacitance is 73.2% at the scan rate of 1 mV s^−1^ (Figure 7c), this means good rate capability.^[^ ^7^ ^]^ And the contribution of capacitance increases with the increase in scan rate (Figure 7d). The rate performance and the corresponding charge‐discharge curves are also shown in Figure 7e,f, respectively. It can be seen that the specific capacity does not significantly decay during the whole process. the QSS N_v_‐VN/C‐SS‐2 //Zn battery also showed a high specific capacity of 217.5 mAh g^−1^ even at 2.5 A g^−1^. Comparing the calculated energy density and power density of the QSS N_v_‐VN/C‐SS‐2//Zn battery with the reported zinc ion battery cathode material^[^ ^38^, ^45^, ^46^, ^47^, ^48^, ^49^ ^]^ (Table S3, Supporting Information), as shown in Figure 7g, the Ragone plot clearly shows a high Zn^2+^ storage capacity of QSS N_v_‐VN/C‐SS‐2//Zn battery. And a high energy density of 278.9 Wh kg^−1^ was obtained at a power density of 94.9 W kg^−1^. When the power density reaches 2375.1 W kg^−1^, it still has a high energy density of 206.5 Wh kg^−1^. In order to better understand the excellent electrochemical performance of QSS N_v_‐VN/C‐SS‐2//Zn battery, we compared the electrochemical impedance spectroscopy (EIS) (Figure 7h) before cycling and after different scan rates. Before the first cycle, the *R*_ct_ value was 398.5 Ω, and after the CV test of 0.4–0.8 mV s^−1^, the *R*_ct_ value decreased to 199.1 Ω, however, after a sweep rate of 1 mV s^−1^, the *R*_ct_ value increases to 703.5 Ω, this indicates that the electrodes were activated during the discharge/charge cycles during the previous cycles. The subsequent increase of *R*_ct_ value illustrates the decay of capacity. Furthermore, the cyclic stability of the QSS N_v_‐VN/C‐SS‐2//Zn battery at 2.5 A g^−1^ was evaluated. The specific capacity remained at 186.5 mAh g^−1^ and the coulombic efficiency was ≈100% after 100 cycles (Figure 7i).

**Figure 8:**
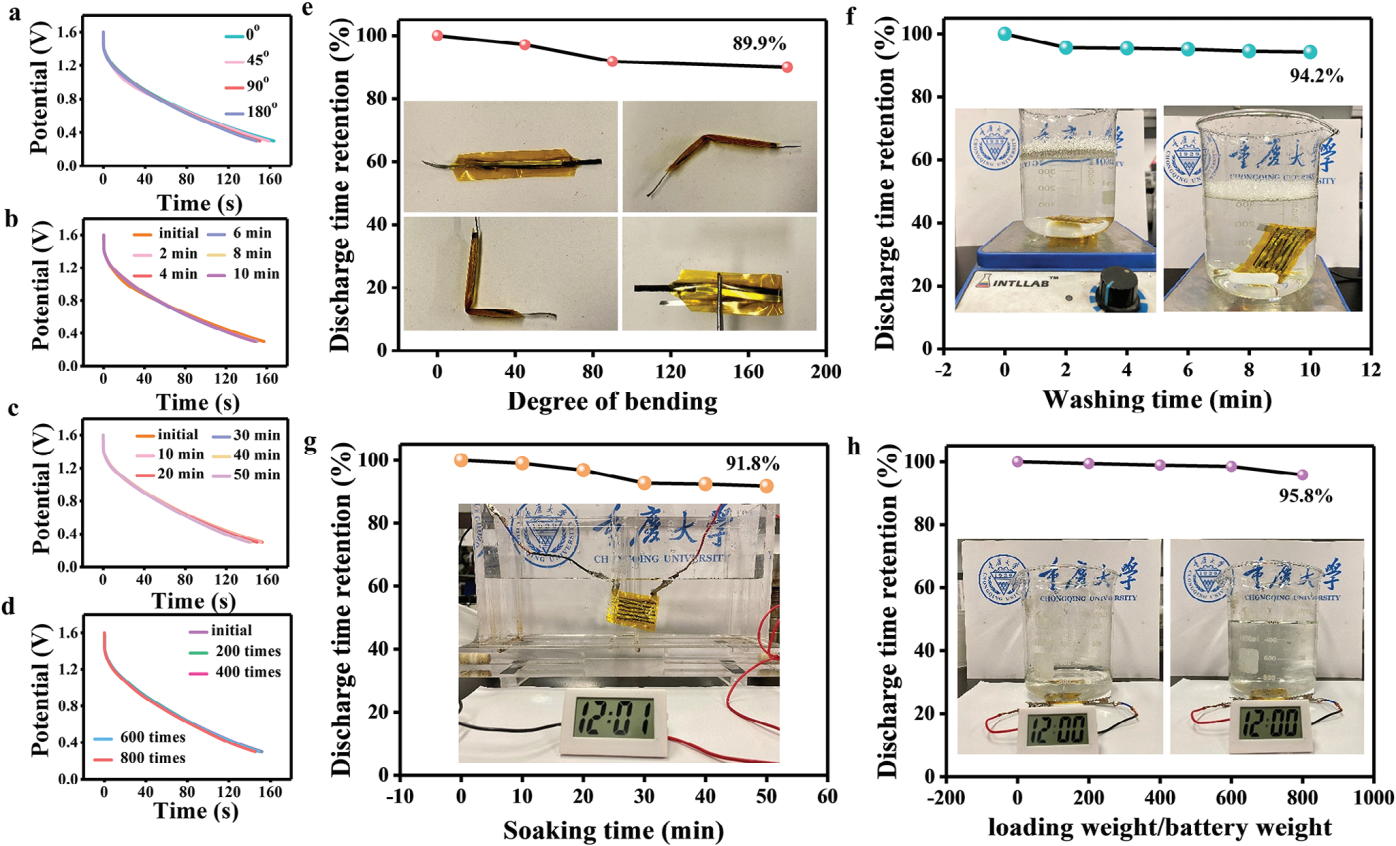
a,e) Charge–discharge curves and actual operation diagram of bending test, b,f) washing test, c,g) soaking test, d,h) weight loading test.

To evaluate the flexibility of FQSS N_v_‐VN/C‐SS‐2//Zn devices and their adaptability in harsh environments, bending, soaking in water, washing, and weighing tests were carried out. As shown in **Figure** **8**a, the flexible quasi‐solid FQSS N_v_‐VN/C‐SS‐2//Zn devices still have almost the same discharge time under different bending degrees, and even under the condition of bending 180°, the discharge time retention rate is as high as 89.9% of that at 0° (Figure 8e). In addition, QSS N_v_‐VN/C‐SS‐2//Zn devices were immersed in water containing detergent to simulate the washing process. After 10 min of washing, the discharge time retention rate of the devices was still as high as 94.2% (Figure 8b,f), showing excellent mechanical robustness. Similarly, the devices also show good water‐blocking ability after being soaked in water for a long time, after continuous soaking for 50 min, the electronic watch can still work normally, and the discharge time retention rate is as high as 91.8% (Figure 8c,g). In addition to good flexibility and water adaptability, the assembled FQSS N_v_‐VN/C‐SS‐2//Zn device also has a good ability to withstand gravity. As shown in Figure 8h, it was found that the FQSS N_v_‐VN/C‐SS‐2//Zn device can bear more than 800 times its own weight while maintaining normal operation of the electronic watch and a long discharge time (Figure 8d). The above experiment shows that the FQSS N_v_‐VN/C‐SS‐2//Zn device can be used under some extreme environmental conditions due to its sufficiently good flexibility and compressive strength.

## 3 Conclusion

In summary, we combined DFT calculation and different test characterization methods to successfully reveal the mechanism of Zn^2+^ ion storage of VN clusters with nitrogen vacancies and C modification as the cathode material of AZIBs. The self‐supporting N_v_‐VN/C‐SS‐2 cathode prepared by the metal–organic framework (MOF) derivation method skillfully embeds the VN clusters containing nitrogen vacancies into C, the excellent structure and the important combination of the nitrogen vacancies enables the electrode to show good electrochemical performance, and the capacity is as high as 257 mAh g^−1^ at 0.2 A g^−1^, simultaneously has the excellent rate performance and the cycle life. Importantly, the formation of vacancies significantly improves the electrochemical reaction reversibility and capacitance contribution, which is also very beneficial to the improvement of performance. The QSS N_v_‐VN/C‐SS‐2//Zn battery also shows excellent rate performance and cycle stability and has high energy density and power density. The assembled flexible quasi‐solid state N_v_‐VN/C‐SS‐2//Zn device also has excellent flexibility, toughness, and outstanding environmental adaptability. Therefore, the results verify that the N_v_‐VN/C‐SS‐2 prepared in this paper provides a new idea to improve the electrochemical performance and reveal the energy storage mechanism of AZIBs, and provides a new insight for the development of FQSS devices with excellent performance even in harsh environments.

## Conflict of Interest

The authors declare no conflict of interest.

## Supporting information

Supporting Information

## Data Availability

The data that support the findings of this study are available on request from the corresponding author. The data are not publicly available due to privacy or ethical restrictions.
