# Supplementary material for: Nitrogen‐Vacancy‐Rich VN Clusters Embedded in Carbon Matrix for High‐Performance Zinc Ion Batteries
Source: Adv Sci (Weinh). 2024 Mar 13;11(19):2308668. doi: 10.1002/advs.202308668 (PMC11109659; doi:10.1002/advs.202308668)
Supplement: Supplementary file 1 — Supporting Information [file ADVS-11-2308668-s001.pdf]

ADVANCED SCIENCE
Open Access

Supporting Information

for *Adv. Sci.*, DOI 10.1002/advs.202308668

Nitrogen-Vacancy-Rich VN Clusters Embedded in Carbon Matrix for High-Performance Zinc Ion Batteries

Youcun Bai, Liang Luo, Wenliang Song, Shuaishuai Man, Heng Zhang and Chang Ming Li**

Supporting information

Nitrogen-Vacancy-Rich VN Clusters Embedded in Carbon Matrix for High Performance Zinc Ion Batteries

Youcun Bai[a], Liang Luo[b], Wenliang Song[c], Shuaishuai Man[d], Heng Zhang[a]*, Chang Ming Li[a]*

[a]Institute for Materials Science and Devices, School of Materials Science & Engineering, Suzhou University of Science & Technology, Suzhou 215011, P. R. China

[b]School of Chemistry and Chemical Engineering, Chongqing University, Chongqing 401331, China

[c]School of Materials and Chemistry, University of Shanghai for Science and Technology, Shanghai 200093, P. R. China

[d]School of Environment and Ecology, Jiangnan University, Wuxi 214122, P.R. China.

*Corresponding author: Chang Ming Li, E-mail: ecmli@swu.edu.cn; Heng Zhang, E-mail: zhangheng@usts.edu.cn

Experimental Section

Materials

Vanadyl acetylacetonate ($C_{10}H_{14}O_5V$) and terephthalic acid ($C_8H_6O_4$) were purchased from Titan Technology. All reagents were AR grade and were not further purified.

Syntheses of precursor

Firstly, 4 mmol vanadyl acetylacetonate and 4 mmol terephthalic acid were dissolved in 40 mL of dimethylformamide by stirring. Secondly, this solution was transferred to

a 50 ml autoclave, and the stainless steel (SS) was dipped in the above solution and maintained at 160 ℃ for 24 h, the resulting stainless steel loaded with active material was then washed several times with ethanol and dried in an oven at 50 ℃ overnight.

Syntheses of N_v-VN/C-SS-2 and other comparative samples

The obtained precursor and $C_3H_6N_6$ (2 g) were placed in two separate positions in a porcelain boat, with $C_3H_6N_6$ placed on the upstream side of the furnace. Subsequently, it was calcined at 800 ℃ in a nitrogen atmosphere for 2 h (5 ℃/min), which is expressed as N_v-VN/C-SS-2. For comparison, the precursors were also calcined 1 h and 3 h, denoted N_v-VN/C-SS-1 and N_v-VN/C-SS-3, respectively.

Preparation of gel electrolyte

First, 2.5 g of ethylene glycol and 1 g of polyvinyl alcohol were added to a 1 M solution of $Zn(CF_3SO_3)_2$. Subsequently, the mixture was stirred at 90 ℃ to form a homogeneous viscous gel, which was then poured into a glass dish and left for 3 h at -20 ℃ to form a clear gel.

Material characterization

The crystal structure and composition of the product were determined by X-ray diffraction (XRD) (Panalytical B.V, PANalytical X'Pert Powder) using Cu Kα radiation. The morphology was characterized by scanning electron microscope (SEM), energy dispersive X-ray (EDS) and element mapping image (Thermo Fisher Ltd, Quattro S). The micro-structure was characterized by transmission electron microscopy (TEM) and selected area electron diffraction (SAED) (Thermo Fisher Scientific CDLtd, Talos F200S). X-ray photoelectron spectroscopy (XPS)

(ESCALAB 250Xi) was performed with an X-ray photoelectron spectroscopy probe using Al K source. Electron spin resonance measurements (EPR, A300) were performed in the X-band with a modulation amplitude of 5.00 G.

Electrochemical measurements

The prepared N_v-VN/C-SS-1, N_v-VN/C-SS-2 and N_v-VN/C-SS-3 is directly used as a working electrode, wherein the loading amount of the active material is about 0.6-1.1 mg cm^{-2}, Zinc foil and glass fiber membrane were used as the anode and separator, respectively, and 3 M $Zn(CF_3SO_3)_2$ as the electrolyte. Cyclic voltammetry (CV) in the range of 0.3-1.6 V and electrochemical impedance spectroscopy (EIS) with amplitude of 5 mV and frequency in the range of 10^{-1}-10^5 Hz were performed on an electrochemical workstation (CHI660E, Chenhua, China). The Galvanostatic Intermittent Titration Technique (GITT), rate capability, and cycle performance tests were carried out on a Land cell test instrument (CT2001A, LAND Electronic Co., China).

The diffusion coefficient (*D*) for zinc ion in the electrode material can be calculated according to the following equation [1]:

$$D=(4/\pi\tau)\, L^2(\Delta E_s / \Delta E_t)^2 \qquad \text{(S1)}$$

Where D is the diffusion coefficient of zinc ions, τ is the relaxation time of the current pulse, L is the diffusion length, which is equal to the thickness of the cathode, and ΔE_s and ΔE_t are the voltage changes caused by the current pulse and constant current charging/discharging, respectively.

DFT Calculations

The theoretical calculations were performed based on spin-polarized density functional theory (DFT) route, implemented by Vienna Ab-initio Simulation Package (VASP) [2] with projector augmented wave (PAW) pseudopotential to tackle the

electron-ion interactions. The exchange and correlation functional were treated with Perdew Burke-Ernzerhof (GGA-PBE) generalized gradient approximation [3]. The cutoff energy was set as 400 eV in all relaxation processes. The convergence criteria were set to 10^{-5} eV for the energy and -0.02 eV/Å for the force. The k-point meshes were set of 3 × 3 × 1, 7 × 7 × 1 for geometry optimization and the electronic structure calculation, respectively. All constructions possess larger than 15 Å vacuum region to minimize the interactions between adjacent image cells.

Adsorption energy (E_{ads}) of Zn^{2+} atom in VN or N-deficient VN was calculated by the following equation:

$$E_{\mathrm{ads}} = E_{\mathrm{Zn}^{2+}/VN(N-deficient\_VN)} - E_{VN(N-deficient\_VN)} - E_{\mathrm{Zn}^{2+}} \quad (1)$$

Where E_{ads} represents the adsorption energy of Zn^{2+} adsorbed on VN or N-deficient_VN. $E_{\mathrm{Zn}^{2+}/VN(N-deficient\_VN)}$, $E_{VN(N-deficient\_VN)}$ and $E_{\mathrm{Zn}^{2+}}$ are the calculated total energy of the Zn^{2+} adsorbed on VN or N-deficient_VN, energy of VN or N-deficient_VN, respectively.

Results and Discussion

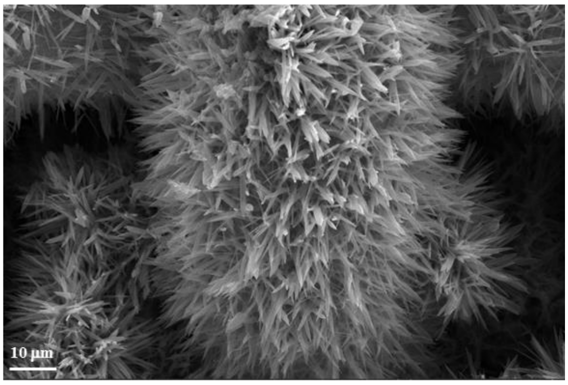

Fig. S1 SEM image of precursor.

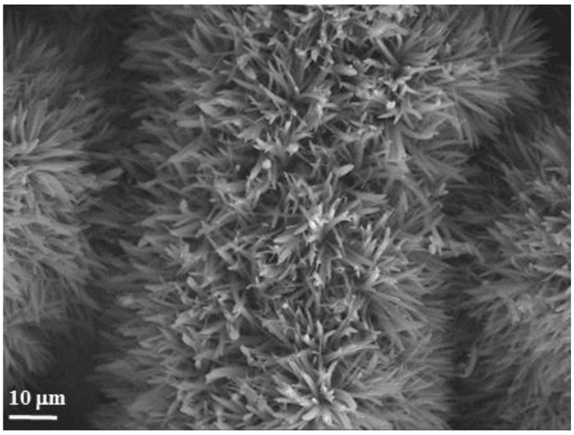

Fig. S2 SEM image of N_v-VN/C-SS-1.

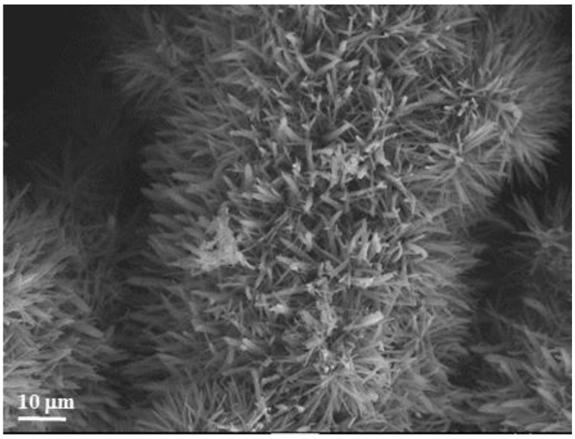

Fig. S3 SEM image of N_v-VN/C-SS-2.

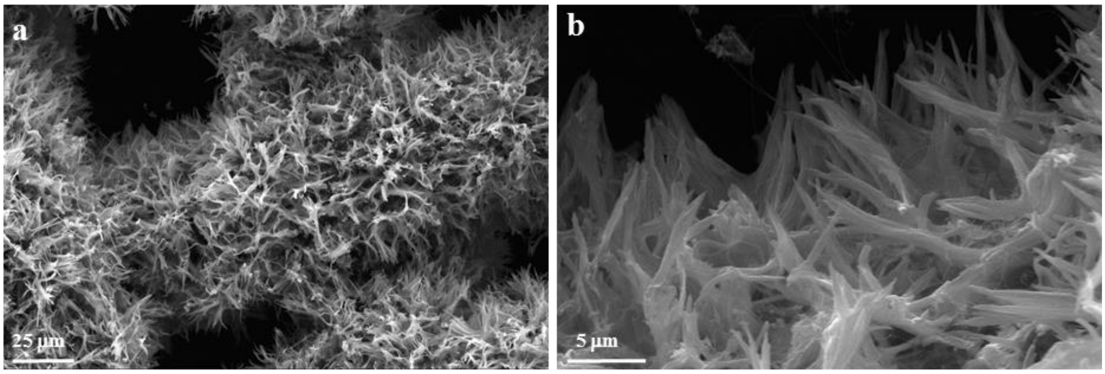

Fig. S4 SEM image of N_v-VN/C-SS-3.

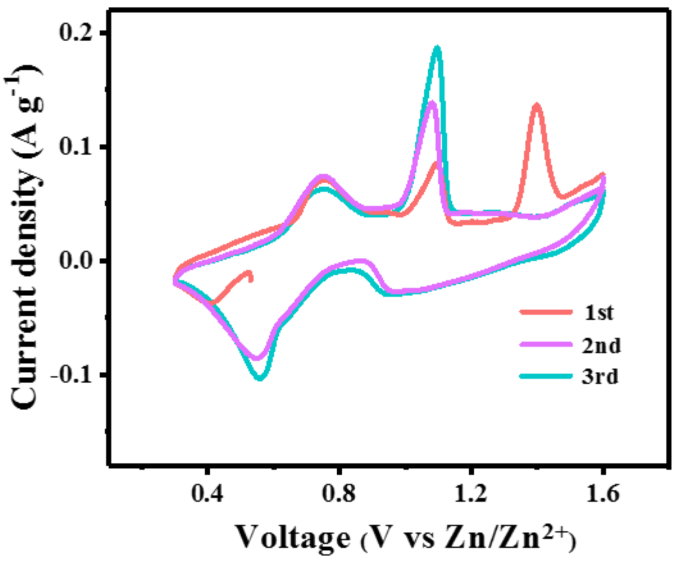

Fig. S5 CV curve of the N_v-VN/C-SS-1.

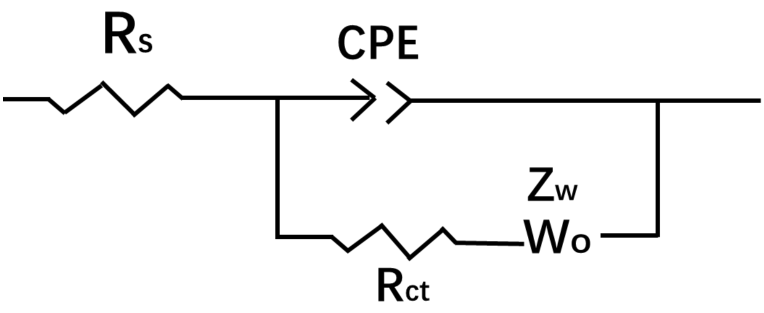

Fig. S6 Equivalent circuit diagram of N_v-VN/C-SS-1, N_v-VN/C-SS-2 and N_v-VN/C-SS-3.

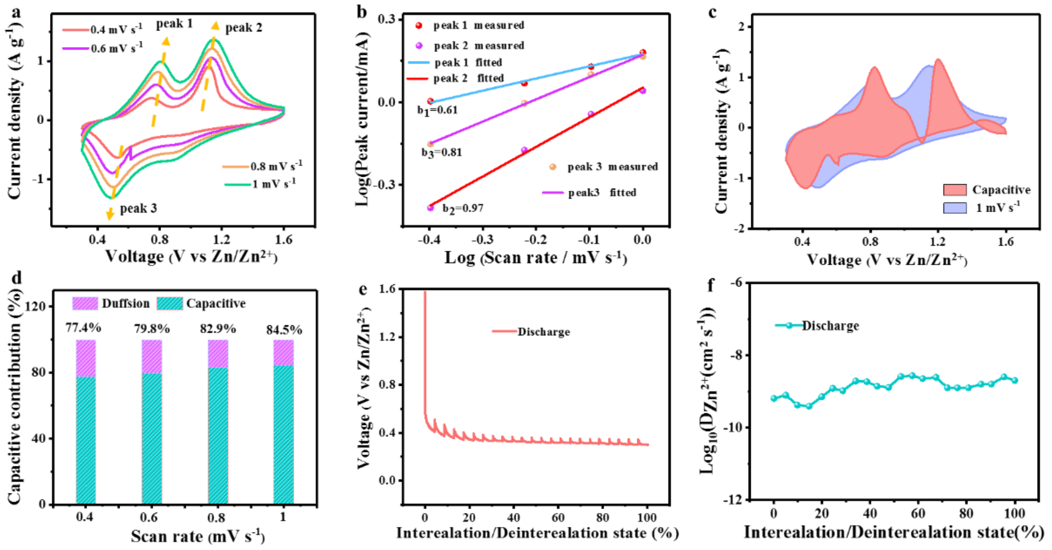

Fig. S7 Electrochemical kinetics for N_v-VN/C-SS-1; CV curves (a), Log (i) versus log (v) plots (b),

capacitive contribution at 1 mV s^{-1} (c), contribution ratio of capacitance and diffusion-controlled

(d), GITT curves (e), Zn^{2+} diffusion coefficient (f).

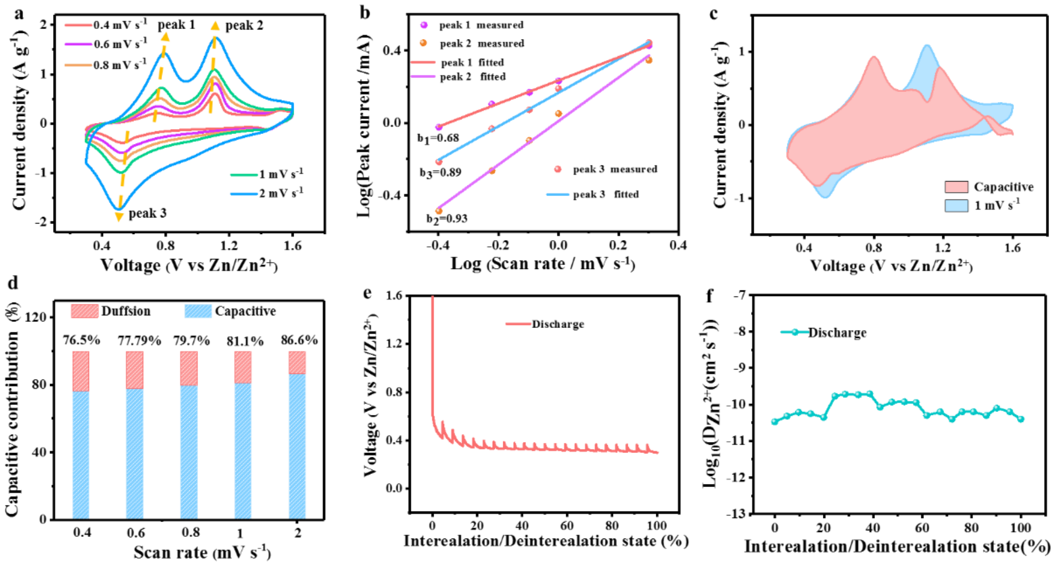

Fig. S8 Electrochemical kinetics for N_V-VN/C-SS-3; CV curves (a), Log (*i*) versus log (*v*) plots (b), capacitive contribution at 1 mV s^{-1} (c), contribution ratio of capacitance and diffusion-controlled (d), GITT curves (e), Zn^{2+} diffusion coefficient (f).

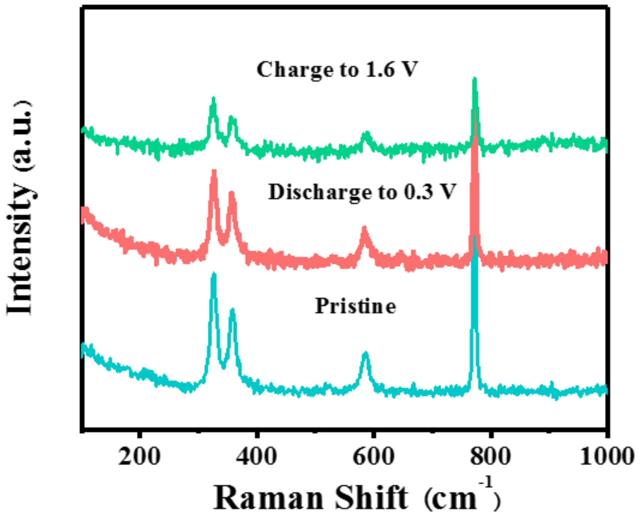

Fig. S9 Raman spectra at different potentials of N_V-VN/C-SS-2.

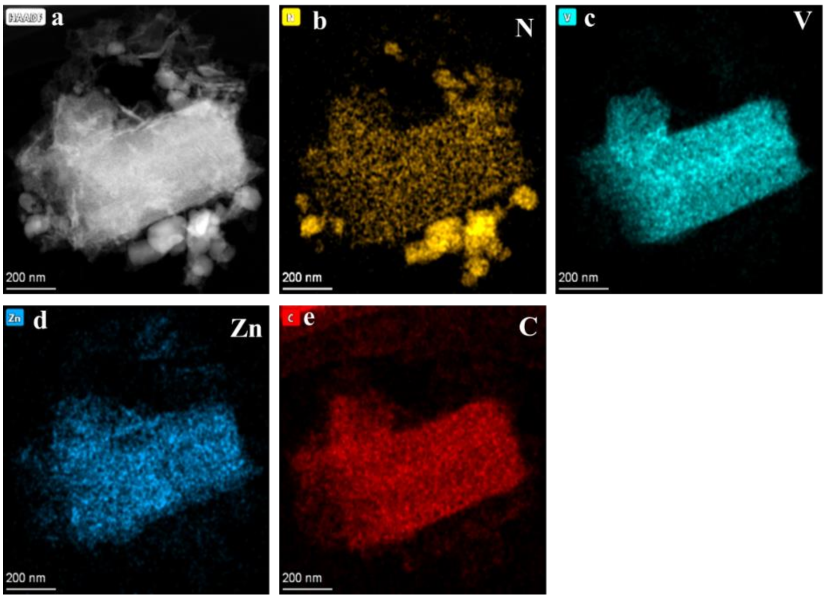

Fig. S10 TEM element mapping of N_v-VN/C-SS-2.

Table S1 Electrochemical performances of some cathode materials in zinc ion storage reported in the previous literatures.

Materials	Current density ($A\ g^{-1}$)	Cycle numbers	Remaining capacity ($mAh\ g^{-1}$)	Ref.
VS_4	2.5	500	110	4
CuVO	0.5	500	92	5
V_2O_5/NaV_6O_{15}	0.5	1000	160	6
VS_2	0.5	200	110.9	7
VSe_2	0.1	500	131.8	8
MnVO	1	500	164	9
P-V_2O_5	2	2000	80	10
$(NH_4)_2V_3O_8/C$	1	2000	135	11
VS_2@VOOH	1.5	350	107.5	12
$Zn_3V_2O_7(OH)_2\cdot 2H_2O$	0.2	300	101	13

**N_v-VN/C-SS-2**	**0.2**	**50**	**257**	**This**
	**4**	**1000**	**227**	**work**

Table S2 EIS fitting parameters of the electrodes based on the corresponding proposed equivalent circuit.

Samples	R_S (Ω)	*CPE*	R_{ct} (Ω)	Z_w (Ω·s1/2)
N_v-VN/C-SS-1	3.908	0.7694	60.52	0.3106
N_v-VN/C-SS-2	2.908	0.5581	33.82	0.3026
N_v-VN/C-SS-3	3.202	0.7106	132.51	0.3502

Table S3 Energy density and power density of some energy storage devices reported in the previous literatures.

**Materials**	**Voltage range (V)**	**Energy density (Wh Kg^{-1})**	**Power density (W Kg^{-1})**	**Ref.**
$Li_{0.45}V_2O_5\cdot 0.89H_2O$	0.3-1.6	137.3	4406	14
C-KVO|O_d	0.1-1.7	272.6	175	15
V_3O_7/V_2O_5	0.2-1.6	135.9	1211	16
$ZMO@Ti_3C_2T_x$	0.8-1.8	230.8	133.7	17
$Zn_3V_2O_7(OH)_2\cdot 2H_2O$	0.2-1.8	214	15	13
E-MoS_2	0.3-1.5	148.2	70.5	18
**N_v-VN/C-SS-2**	**0.3-1.6**	**278.9**	**94.9**	**This work**
		**206.5**	**2375.1**	

References:

1. Y. Cai, R. Chu, Z. K. Kou, H. Ren, D. Yuan, S. Z. Huang, S. Kumar, V. Verma, P. Amonpattaratkit, M. Srinivasan, Boosting Zn-ion storage performance of

bronze-type VO_2 via Ni-mediated electronic structure engineering, ACS Appl. Mater. Interfaces 12 (2020) 36110-36118.

2. Kresse, G.; Hafner, J. Ab Initio Molecular Dynamics for Open Shell Transition Metals. Phys. Rev. B: Condens. Matter Mater. Phys. 1993, 48, 13115−13118.
3. C. He, R. Wang, D. Xiang, X. Li, L. Fu, Z. Jian, J. Huo, S. Li, Appl. Surf. Sci., 509 (2020) 145392.
4. Q. C. Zhu, Q. Xiao, B. W. Zhang, Z. C. Yan, X. Liu, S. Chen, Z. F. Ren, Y. Yu, VS_4 with a chain crystal structure used as an intercalation cathode for aqueous Zn-ion batteries, J. Mater. Chem. A, 2020, 8,10761.
5. L. L. Chen, Z. H. Yang, J. Wu, H. Z. Chen, J. L. Meng, Energy storage performance and mechanism of the novel copperpyrovanadate $Cu_3V_2O_7(OH)_2{\cdot}2H_2O$ cathode for aqueous zinc ion batteries, Electrochimica Acta 330 (2020) 135347.
6. Y. Ran, P. Hong, J. Ren, B. S. Wang, M. J. Xiao, Y. H. Chen, X. C. Xiao, Y. D. Wang, V_2O_5/NaV_6O_{15} nanocomposites synthesizedby molten salt method as a highperformances cathode material for aqueous zinc-ion batteries, Nanotechnology 33 (2022) 115402.
7. P. He, M. Y. Yan, G. B. Zhang, R. M. Sun, L. N. Chen, Q. Y. An, L. Q. Mai, Layered VS_2 nanosheet-based aqueous Zn ion battery cathode, Adv. Energy Mater. (2017) 1601920.
8. Z. Y. Wu, C. J. Lu, Y. N. Wang, L. Zhang, L. Jiang, W. C. Tian, C. L. Cai, Q. F. Gu, Z. M. Sun, L. F. Hu, Ultrathin VSe_2 nanosheets with fast ion diffusion and robust structural stability for rechargeable zinc-ion battery cathode, Small 16 (2020) 2000698.

9. T. H. Wu, Y. M. Li, K. Y. Ni, T. K. Li, W. S. Lin, Vanadium oxides obtained by chimie douce reactions: The influences of transition metal species on crystal structures and electrochemical behaviors in zinc–ion batteries, Journal of Colloid and Interface Science 608 (2022) 3121–3129.
10. Y. C. Ding, Y. Q. Peng, W. Y. Chen, Y. J. Niu, S. G. Wu, X. X. Zhang, L. H. Hu, V-MOF derived porous V_2O_5 nanoplates for high performance aqueous zinc ion battery, Applied Surface Science 493 (2019) 368–374.
11. H. M. Jiang, Y. F. Zhang, L. Xu, Z. M. Gao, J. Q. Zheng, Q. S. Wang, C. G. Meng, J. Wang, Fabrication of $(NH_4)_2V_3O_8$ nanoparticles encapsulated in amorphous carbon for high capacity electrodes in aqueous zinc ion batteries, Chemical Engineering Journal 382 (2020) 122844.
12. X. M. Pu, T. B. Song, L. B. Tang, Y. Y. Tao, T. Cao, Q. J. Xu, H. M. Liu, Y. G. Wang, Y. Y. Xia, Rose-like vanadium disulfide coated by hydrophilic hydroxyvanadium oxide with improved electrochemical performance as cathode material for aqueous zinc-ion batteries, Journal of Power Sources 437 (2019) 226917.
13. C. Xia, J. Guo, Y. J. Lei, H. F. Liang, C. Zhao, H. N. Alshareef, Rechargeable aqueous zinc-ion battery based on porous framework zinc pyrovanadate intercalation cathode, Adv. Mater. (2017) 1705580.
14. Y. X. Tong, X. M. Li, S. D. Su, J. Z. Li, J. Z. Fang, B. Liang, J. H. Hou, M. Luo, Hydrated lithium ions intercalated V_2O_5 with dual-ion synergistic insertion mechanism for high-performance aqueous zinc-ion batteries, Journal of Colloid and Interface Science 606 (2022) 645-653.
15. W. Yang, L. B. Dong, W. Yang, C. J. Xu, G. J. Shao, G. X. Wang, 3D Oxygen-Vacancyive Potassium Vanadate/Carbon Nanoribbon Networks as

High-Performance Cathodes for Aqueous Zinc-Ion Batteries, Small Methods 2019, 1900670.

16. H. Z. Chen, L. L. Chen, J. L. Meng, Z. H. Yang, J. Wu, Y. Rong, L. Deng, Y. D. Shi, Synergistic effects in V_3O_7/V_2O_5 composite material for high capacity and long cycling life aqueous rechargeable zinc ion batteries, Journal of Power Sources 474 (2020) 228569.
17. M. J. Shi, B. Wang, Y. Shen, J. T. Jiang, W. H. Zhu, Y. J. Su, M. Narayanasamy, S. Angaiahc, C. Yan, Q. Peng, 3D assembly of MXene-stabilized spinel ZnMn2O4 for highly durable aqueous zinc-ion batteries, Chemical Engineering Journal 399 (2020) 125627.
18. H. F. Li, Q. Yang, F. N. Mo, G. J. Liang, Z. X. Liu, Z. J. Tang, L. T. Ma, J. Liu, Z. C. Shi, C. Y. Zhi, MoS_2 nanosheets with expanded interlayer spacing for rechargeable aqueous Zn-ion batteries, Energy Storage Mater. 19 (2019) 94–101.
